# Intestinal microbiota changes in early life of very preterm infants with bronchopulmonary dysplasia: a nested case–control study

**DOI:** 10.3389/fmicb.2025.1632412

**Published:** 2025-07-07

**Authors:** Tao Ning, Xiaoxue Shan, Xiaowen Zhuang, Bingjie Li, Yuhan Zhang, Tianyu Chen, Song Peng, Han Lu, Qiong Xue, Huaiyu Yan, Yimei Ren, Shudong Cui, Xiaoqing Chen

**Affiliations:** ^1^Department of Pediatrics, The First Affiliated Hospital with Nanjing Medical University, Nanjing, China; ^2^Department of Pediatrics, The Affiliated Suqian Hospital of Xuzhou Medical University, Suqian, China

**Keywords:** intestinal/gut microbiota, microbiota dysbiosis, preterm infant, bronchopulmonary dysplasia, high-throughput sequencing

## Abstract

**Background and aim:**

Bronchopulmonary dysplasia (BPD) is one of the most important complications of very preterm infants. This study was to investigate changes in the intestinal microbiota of very preterm infants with BPD.

**Methods:**

We enrolled 50 very preterm infants at the gestational age of 24+^0^–31+^6^ weeks, categorizing them into the BPD group and control group, and fecal samples were collected on days 1, 7, 14, 21, and 28, respectively. Finally, 30 preterm infants were left after excluding 20 preterm infants. We tested and analyzed 16S rDNA of bacteria and short-chain fatty acids (SCFAs) within the feces.

**Results:**

The BPD group possessed a higher abundance of *Ureaplasma urealyticum* (UU) and a lower abundance of *Bacteroidota* than the control on day 1. The differences in intestinal microbiota were reduced on days 7 and 14, and no difference in SCFAs existed on day 14. New differences emerged over time, with a significant decrease of *Veillonella dispar* in the BPD group than in the control group on day 28, which showed a continuous decline in the BPD group over time.

**Conclusion:**

Intestinal microbiota dysbiosis existed in very preterm infants with BPD. The increased abundance of UU on day 1 and the decrease of *Veillonella dispar* on day 28 might increase the risk of BPD.

## 1 Introduction

The enhanced survival rates of extremely and very preterm infants have led to an increase in developmental complications, BPD (Thébaud et al., 2019), affecting infants whose lungs remain in the canalicular or saccular stages. This condition occurs in 15–25% of very preterm infants and affects up to 60% of extremely preterm infants (Pierro et al., 2022). A growing number of animal experiments and human observational studies have shown that BPD is accompanied by changes in intestinal microbiota and its metabolites (Zhang et al., 2022). However, the changes in intestinal microbiota in very preterm infants with BPD and their underlying mechanisms have not been totally clarified. Our purpose in conducting this study was to gain a deep insight into the dynamic changes and differences in intestinal microbiota between the BPD group and the control group.

## 2 Methods

### 2.1 Ethics approval and consent

This study, which was conducted in the Level-III NICU at the First Affiliated Hospital of Nanjing Medical University in Nanjing, China, was approved by the Ethics Committee of the hospital (2022-SR-720) and conducted in accordance with ethical standards as laid down in the 1964 Declaration of Helsinki. All parents provided written informed consent before the study began.

### 2.2 Study design and participants

**Figure 1:**
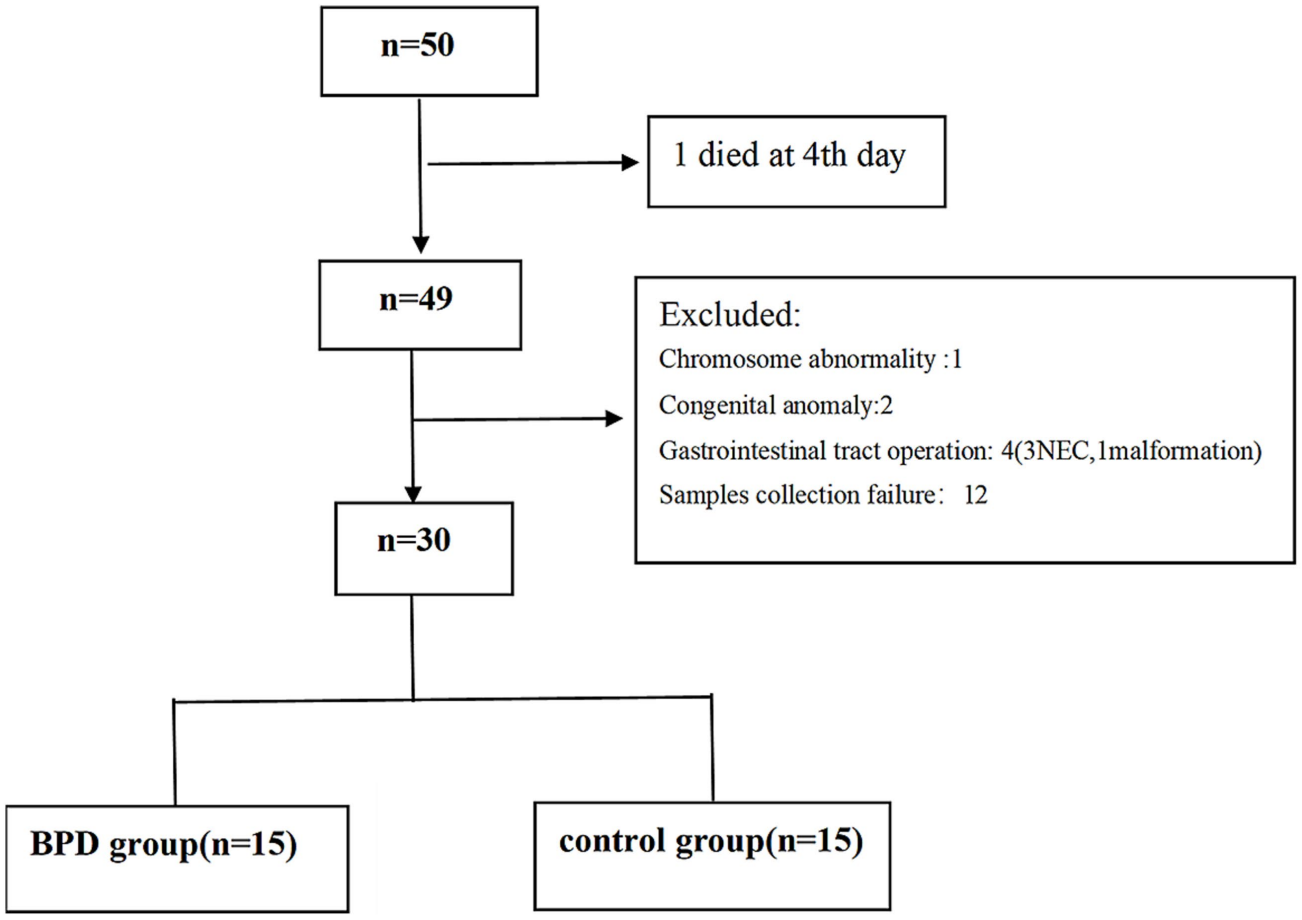
CONSORT flow chart for the enrolled preterm infants.

Preterm infants at the gestational age of 24+^0^–31+^6^ weeks were eligible for inclusion. The exclusion criteria included major congenital anomalies, gastrointestinal tract operations, mothers who were alcohol drinkers or smokers, and those who failed to submit informed consent. We enrolled 50 very preterm infants from February 2023 to September 2023. After applying the exclusion criteria, 30 infants remained in the study. The eligible were divided into the BPD group (*n* = 15) and control group (*n* = 15), as shown in Figure 1.

The participants were at the gestational age of 24+^0^–31+^6^ weeks and had a birth weight of 520–2,050 g. The antibiotics used for preterm infants were a combination of ampicillin and cefoperazone-sulbactam, according to the treatment practices of the local hospital.

The diagnostic criteria for BPD, as proposed by the National Institute of Child Health and Human Development in 2018, applied to preterm infants with gestational age less than 32 weeks. These infants required more than 3 days of oxygen therapy to keep arterial oxygen saturation between 90 and 95% at a postmenstrual age of 36 weeks. Additionally, these criteria necessitated the presence of persistent parenchymal lung disease confirmed by imaging.

### 2.3 Sample collection

A collection of fecal samples was performed on days 1, 7, 14, 21, and 28, respectively. When the feces were discharged, the researchers collected the samples promptly using a sterile sampling spoon. The samples were subsequently stored in sterile containers at −80°C for 30 min. We collected 139 stool samples and then delivered them on dry ice to BGI Genomics in Wuhan, China, for examination. With four samples that failed to be tested because of low concentration of DNA, the data from 135 fecal samples were eligible for subsequent analyses. We reserved a portion of the fecal samples collected on day 14 for the analysis of SCFAs.

### 2.4 16S rDNA analytical procedures

We applied high-throughput sequencing methods to investigate the 16S rDNA of the fecal bacteria. Approximately 0.1 g of feces were placed into a centrifuge tube, and an appropriate amount of lysis buffer (Qiagen, Germantown, MD, USA) was added. A suitable amount of glass beads (Thermo Fisher Scientific, Waltham, MA, USA) was then added to the sample, which was vortexed vigorously for 3–5 min to mechanically disrupt the bacterial cell walls and incubated at 56°C for a duration of 30–60 min thereafter. The samples were heated to 95°C for 10 min and centrifuged at 12,000 rpm for 10 min. The DNA pellets were washed with cold 70% ethanol (Thermo Fisher Scientific). After air drying to remove residual ethanol, the DNA pellets were dissolved in an appropriate volume of nuclease-free water. DNA concentration and purity were measured using a spectrophotometer (Thermo Fisher Scientific) by checking the A260/A280 ratio.

The variable regions V3 and V4 of the 16S rDNA were amplified using polymerase chain reaction (PCR) with the corresponding fusion primers 338F (5′ ACTCCTACGGGAGGCAGCAG 3′) and 806R (5′ GGACTACHVGGGTWTCTAAT 3′). The PCR reaction system was set up using 30 ng of DNA samples and the specified primers. Agencourt AMPure XP magnetic beads (A&D Technology, Tokyo, Japan) were applied to the purification of PCR amplification products to be dissolved in Elution Buffer (Thermo Fisher Scientific). Labels were attached to complete the library preparation. The Agilent 2100 Bioanalyzer (Agilent Technologies, Beijing, China) was used to measure the fragment range and concentration of the library. Based on the size of the inserted fragments, the qualified libraries were then sequenced on an Illumina MiSeq (Illumina, Inc., San Diego, CA, USA) platform.

After the raw data was filtered, high-quality clean reads were obtained. The primers and adapter sequences of the reads matching the primers were removed to obtain the fragments of the target region with Cutadapt (v2.6, Biotronik, Berlin, Germany). When the average value was lower than 20 over a 30 bp sliding window, the terminal sequence of reads was truncated; reads truncated to less than 75% of their original length, as well as those with ambiguous bases or low complexity, were removed.

Thereafter, clean data were prepared for the subsequent analysis. The reads were spliced into tags based on overlapping regions. When the paired-end reads overlapped, a consensus sequence was generated using FLASH (v1.2.11; Biotronik). The tags were clustered to generate operational taxonomic units (OTUs) using USEARCH (v7.0.1090; Qiagen) according to 97% sequence similarity. Chimeras were filtered using UCHIME (v4.2.40; University of California, Santa Cruz, CA, USA).

OTU representative sequences were matched against the database for taxonomic annotation using the RDP classifier (v1.9.1; University of Michigan, Ann Arbor, MI, USA). With the database compared, OTUs were taxonomically classified at phylum, class, order, family, genus, and species levels. We selected the top 10 most abundant species, defined them as key species, and displayed their average relative abundance in each group along with the significance of the differential tests.

### 2.5 SCFAs analytical procedures

Ten samples were selected from the BPD group and ten from the control group. A test was performed on the SCFAs (acetate, butyrate, propionate, valerate, caproate, isobutyrate, and isovalerate) in these samples at the BGI. The feces-derived SCFAs were quantitatively detected by LC–MS/MS using a detection platform equipped with a Waters Iclass-AB Sciex 6,500 LC/MS system (Waters Corporation, Middleton, MA, USA).

A fecal sample (25 mg) was placed into a centrifuge tube, with the addition of 400 uL of methanol-acetonitrile mixture (Thermo Fisher Scientific) and magnetic beads to break it, followed by a 2-min centrifugation performed at 25,000 rpm. For the standard curve, seven types of SCFA mixtures were used for the gradient dilution. To the 20ul sample and standard curve, 60ul cold MeOH/ACN (2:1) (Aslan Chemical Co., Ltd., Shanghai, China) was added, followed by 5 min of shaking and 4 h of precipitation at −20°C. Subsequently, the sample was centrifuged at 20,000 rpm at 4°C for 15 min, and 40 μL of the supernatant was extracted from the EP tube (Eppendorf, Hamburg, Germany). For derivatization, 20 μL of 200 mM 3-NPH (Waters Corporation) and 20 μL of a 120 mM EDC-6% pyridine mixture (Thermo Fisher Scientific) were added to the EP tube. The tubes were then incubated in a metal bath and stirred at 40°C for 30 min. After incubation, the tube was cooled to room temperature on ice before being centrifuged briefly. Finally, 80 μL of 1000D internal standard (solvent: 10% ACN) (Agilent Technologies) was added to the derivatized EP tube and mixed well. To the filter plate were added 90 uL H2O and 90 uL derivatized sample before 5-min centrifugation at 3,000 rpm at 4°C. From a new 96-well plate, 90 uL filtered liquid was collected and 10 uL loaded onto the LC–MS.

### 2.6 Statistical analysis

We used Statistical Package for the Social Sciences (SPSS version 26; SPSS Inc., Armonk, NY, USA) to analyze the clinical data. Normally distributed numerical variables of the two groups were compared using Student’s independent sample *t-*test, expressed as mean and standard deviation (SD), while the Wilcoxon rank sum test was performed to compare variables that did not follow a normal distribution, expressed as median and interquartile range (IQR). Counts and percentages were used for categorical variables, and Fisher’s exact test was used to test for differences in the data. A *p*-value less than 0.05 was deemed statistically significant. A difference analysis was conducted to test differences in the relative abundance of bacteria. The Mann–Whitney U test was used to compare two groups, and the Kruskal–Wallis test was used to compare more than two groups. Alpha diversity was measured using Shannon and Simpson indices. Beta diversity analysis was performed to evaluate the sample differences in species complexity. Additionally, PICRUSt2 (v2.3.0, University of California) was used to predict the functional abundance of the microbial communities based on marker gene sequencing profiles. In the analysis of SCFA differences between the two groups, the ratio analysis and *t-*test were performed to evaluate them.

## 3 Results

### 3.1 Participant characteristics

The gut microbiota we studied was during the first 28 days after birth; therefore, the clinical data related to the microbiota we collected were mostly during the first 28 days after birth. Subsequently, a comparison was made between the clinical data of the two groups, and the results indicated a lower gestational age in the BPD group than in the control group (*p* = 0.007; Table 1), but no significant difference in birth weight between the two groups. As shown in Table 1, the BPD group had a higher rate of endotracheal intubation (*p* = 0.017) and a lower Apgar score at the time point of 5 min (*p* = 0.008) at birth. Moreover, in the BPD group, a higher rate of invasive ventilation (*p* = 0.002), longer duration of invasive ventilation (*p* = 0.002), and longer duration of non-invasive ventilation (*p* < 0.001) were observed. The BPD group also received a longer administration of antibiotics (*p* = 0.003) during the 28 days after birth, and it took a longer time for them to initiate enteral nutrition (*p* = 0.002) and terminate parenteral nutrition (PN) (*p* < 0.001). Notably, the BPD group had more cases of maternal chorioamnionitis (4 vs. 1) and more infants diagnosed with *Ureaplasma urealyticum* (UU) infection than the control group (8 vs. 0; *p* = 0.002).

**Table 1:** Demographic characteristics and clinical data of all very preterm infants.

Demographic characteristics and clinical data	BPD (*n* = 15)	Control (*n* = 15)	*p* value
Male gender, *n* (%)	7 (46.7)	9 (60)	0.715
Gestational age, mean (SD), week	28 (2.2)	31(0.6)	0.007
Birth weight, mean (SD), g	1,076 (360)	1,606 (272)	0.678
Endotracheal intubation, *n* (%)	6 (40)	0 (0)	0.017
Apgar score at 5 min, median (IQR)	8(8–9)	9.0 (9–10)	0.008
Mother’s age, mean (SD), year	30.5 (3.8)	32.8 (5.9)	0.052
Antenatal steroid use, *n* (%)	7 (46.7)	11 (73.3)	0.264
Maternal chorioamnionitis, *n* (%)	4 (26.7)	1 (6.7)	0.330
C-cesarean, *n* (%)	9 (60)	10 (66.7)	1.000
Duration of prenatal antibiotics, median (IQR), days	2.0 (0–8)	1 (0–3)	0.486
Postnatal antibiotic use, *n* (%)	14 (93.3)	15 (100)	1.000
Duration of antibiotics before day 28, mean (SD), day	14.4 (8.7)	8.5 (2.8)	0.003
UU infection, *n* (%)	8(53.3)	0(0)	0.002
Initiation of feeding, mean (SD), day	6.4(4.0)	2.5(1.4)	0.002
Duration of PN, mean (SD), day	50.93(32.3)	15.3(8.5)	<0.001
Breast and mixed feeding, *n* (%)	6 (40)	12(80)	0.060
Invasive ventilation, *n* (%)	10 (66.7)	1(6.7)	0.002
Duration of invasive ventilation before day 28, median (IQR), day	3(0–13)	0(0–0)	0.002
Duration of non-invasive ventilation before day 28, mean (SD), day	26.1(17.7)	3.67 (2.3)	<0.001
Duration of oxygen inhalation before day 28, median (IQR), day	1.0 (0–15.0)	12.0 (5.0–17.0)	0.067

Data were expressed as number (%), where Fisher’s exact test was used; as median (IQR), where the Wilcoxon rank sum test was used; or as mean (SD), where Student’s independent sample *t*-test was used for comparison.

BPD, bronchopulmonary dysplasia; SGA, small for gestational age; IQR, interquartile range; SD, standard deviation; UU, *Ureaplasma urealyticum*; PN, parenteral nutrition.

As indicated in Table 2, the BPD group had more periventricular leukomalacia (PVL), intraventricular hemorrhage (IVH), and hemodynamically significant patent ductus arteriosus (hsPDA) than the control group, although there were no statistically significant differences. Greater retinopathy of prematurity (ROP) (*p* = 0.042) was observed in the BPD group, along with a higher number of nosocomial infections.

**Table 2:** Clinical outcomes of all very preterm infants.

Outcomes	BPD (*n* = 15)	Control (*n* = 15)	*p* value
BPD, *n*	Grade I:12 Grade II:1 Grade III:2		
IVH, *n*	Grade I-II:6 Grade III-IV:3	Grade I-II:5 Grade III-IV:1	1.000 0.598
PVL, *n*	3	0	0.224
ROP, *n*	5	0	0.042
Pneumothorax, *n*	1	0	1.000
hsPDA, *n*	2	0	0.483
NEC, *n*	1	1	1.000
Late-onset sepsis, *n*	6	0	0.017
Nosocomial pneumonia, *n*	4	0	0.100
Nosocomial fungemia, *n*	4	0	0.100

BPD was diagnosed in accordance with the criteria of the NICHD in 2018.

BPD, bronchopulmonary dysplasia; IVH, intraventricular hemorrhage; PVL, periventricular leukomalacia; ROP, retinopathy of prematurity; PDA, patent ductus arteriosus; NEC, neonatal necrotizing enterocolitis.

### 3.2 Species accumulation curve, OTUs detected at different times

**Figure 2:**
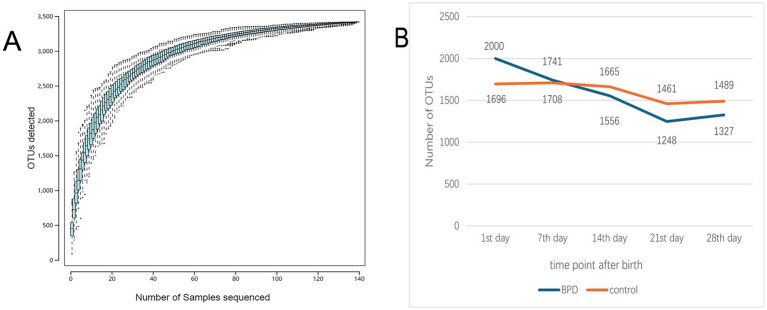
The species accumulation curve flattened out as the number of samples increased to indicate that the sampling quantity was adequate **(A)**, and the number of OTUs was compared between the two groups **(B)**.

As shown in Figure 2A, the analysis of species accumulation produced a flat curve at the terminal, which indicated adequate sampling quantity. Through analysis of OTUs in the two groups, we observed that the OTUs in BPD group were more abundant on postnatal day 1 but subsequently showed a declining trend, falling below the level of the control after one week of birth. In contrast, the OTU count in the control remained relatively stable (Figure 2B).

### 3.3 Intestinal microbiota comparison of all specimens in the BPD and control groups

**Figure 3:**
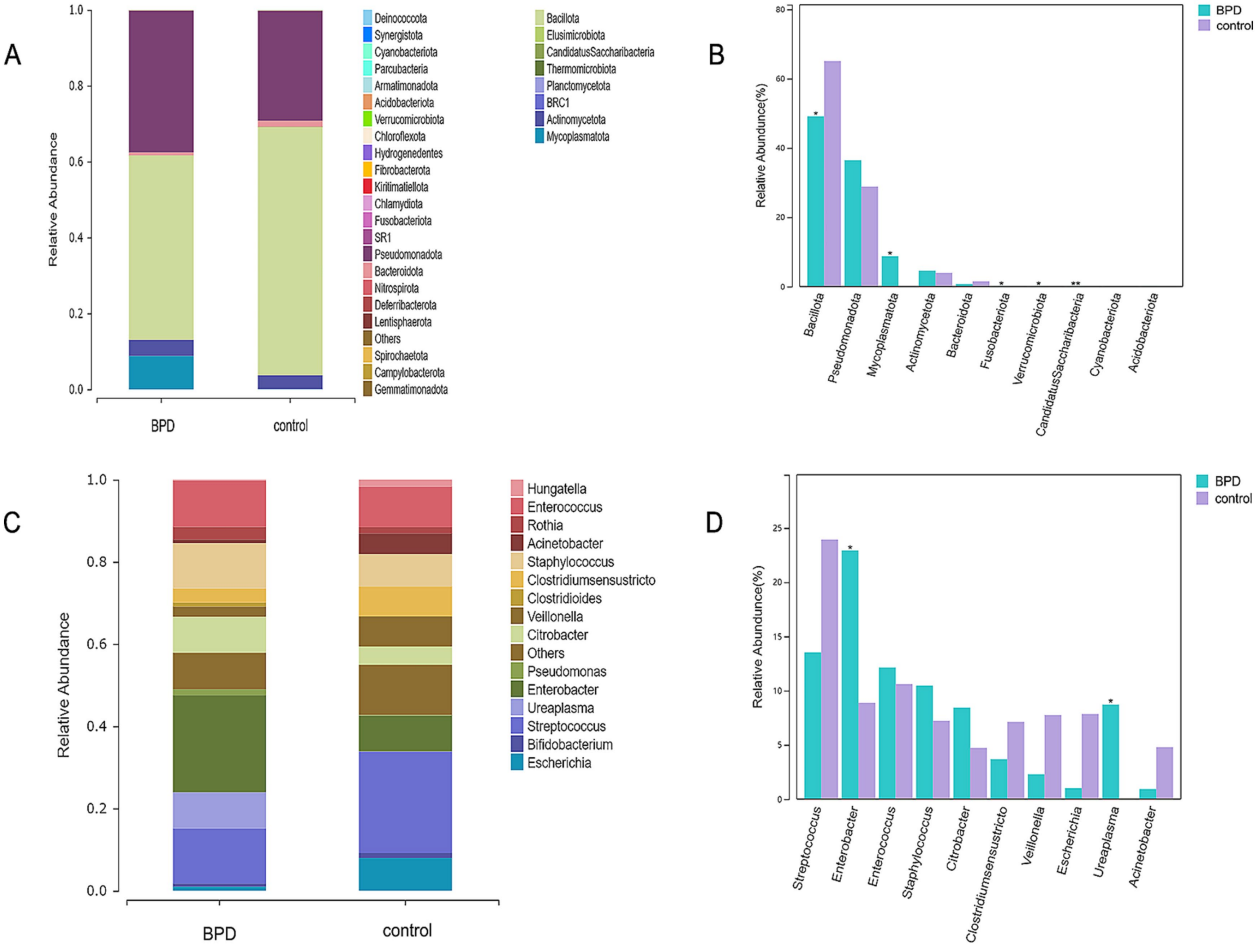
Dominant phyla in the BPD group within 28 days after birth **(A)**, higher abundance of *Mycoplasmatota* and lower abundance of *Bacillota* in the BPD group **(B)**, dominant genera in the BPD group **(C)**, and the BPD group had a higher abundance of *Enterobacter* and *Ureaplasma* **(D)**.

At 28 days after birth, the dominant phyla in the BPD group were *Bacillota*, *Pseudomonadota*, *Mycoplasmatota*, *Actinomycetota*, and *Bacteroidota*, whereas those in the control group were *Bacillota*, *Pseudomonadota*, *Actinomycetota*, and *Bacteroidota* (Figure 3A). The BPD group had a higher abundance of *Mycoplasmatota* (*p* = 0.013) and a lower abundance of *Bacillota* than the control *group* (*p* = 0.029; Figure 3B). In the 28 days after birth, the dominant genera were *Enterobacter*, *Streptococcus*, *Enterococcus*, *Staphylococcus*, and *Ureaplasma* in the BPD group, whereas those in the control group were *Streptococcus*, *Enterococcus*, *Enterobacter*, *Escherichia*, and *Veillonella* (Figure 3C). A significant increase in the relative abundance of *Enterobacter* (*p* = 0.020) and *Ureaplasma* (*p* = 0.010) was observed in the BPD group compared to that in the control group (Figure 3D).

### 3.4 Longitudinal comparison of microbiota in the BPD and control groups, respectively

**Figure 4:**
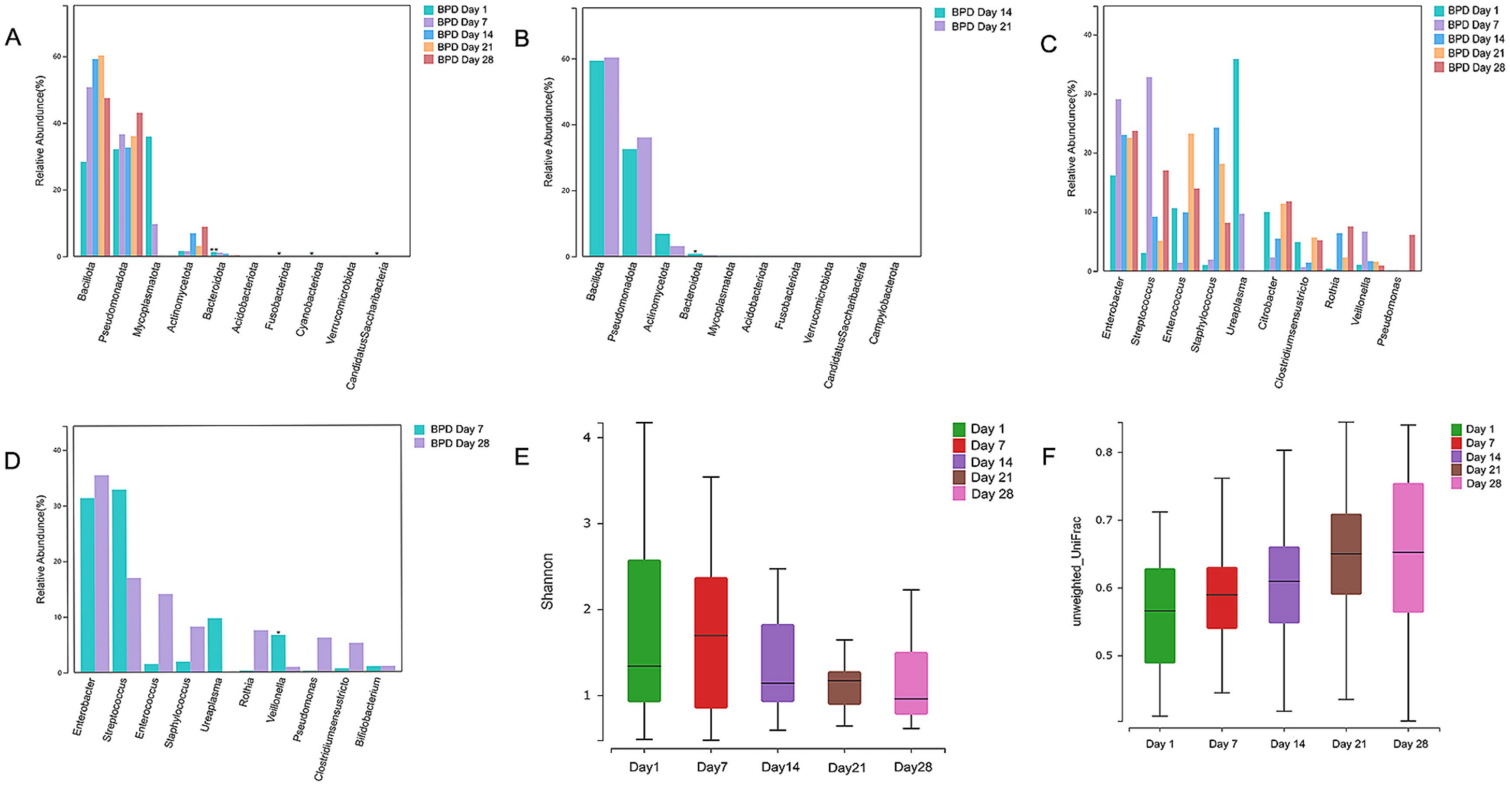
*Bacteroidota*, *Veillonella*, and Shannon indices continuously declined in the BPD group at 28 days after birth. Comparisons of phyla between days 1, 7, 14, 21, and 28 in the BPD group showed a continuous decline trend of *Bacteroidota* from birth to day 21 **(A)**, a significant decline in *Bacteroidota* from days 14 to 21 in the BPD group **(B)**, a transient increase from days 1 to 7, and a significant decline from days 7 to 28 of *Veillonella*, and the continuous high abundance of *Enterobacter* in the BPD group **(C)**, a significant decline from days 7 to 28 of *Veillonella* in the BPD group **(D)**, a Shannon index chart on days 1, 7, 14, 21, and 28 in the BPD group **(E)**, a beta diversity chart on days 1, 7, 14, 21, and 28 in the BPD group **(F)**.

From the intestinal microbiota compared at different time intervals in the BPD group, we found that *Bacteroidota* showed a continuous declining trend from birth to day 21 and a significant decline from day 14 to day 21 (*p* = 0.015; Figures 4A,B). *Mycoplasmatota* was the dominant phylum on day 1 in the BPD group, followed by a rapid decrease, but without a statistical difference. As shown in Figures 4C,D, a transient increase was observed in *Veillonella* from days 1 to 7, followed by a significant decline from days 7 to 28 (*p* = 0.023) in the BPD group, the main species being *Veillonella dispar*. From days 1 to 7, the abundance of *Enterobacter* showed an increasing trend and then remained high, the main species being *Enterobacter hormaechei*. In contrast, in the case of *Veillonella*, there was no significant difference between the five time points, and an increasing trend was observed from days 14 to 28 in the control group (*p* = 0.051). *Enterobacter* decreased significantly from days 1 to 7 in the control (*p* = 0.030), followed by a further decline from days 7 to 14 (*p* = 0.042). Additionally, the Shannon index decreased with time, but there was an upward trend in the beta diversity in the BPD group (Figures 4E,F).

### 3.5 Horizontal comparison of microbiota between the BPD and control groups at different times

**Figure 5:**
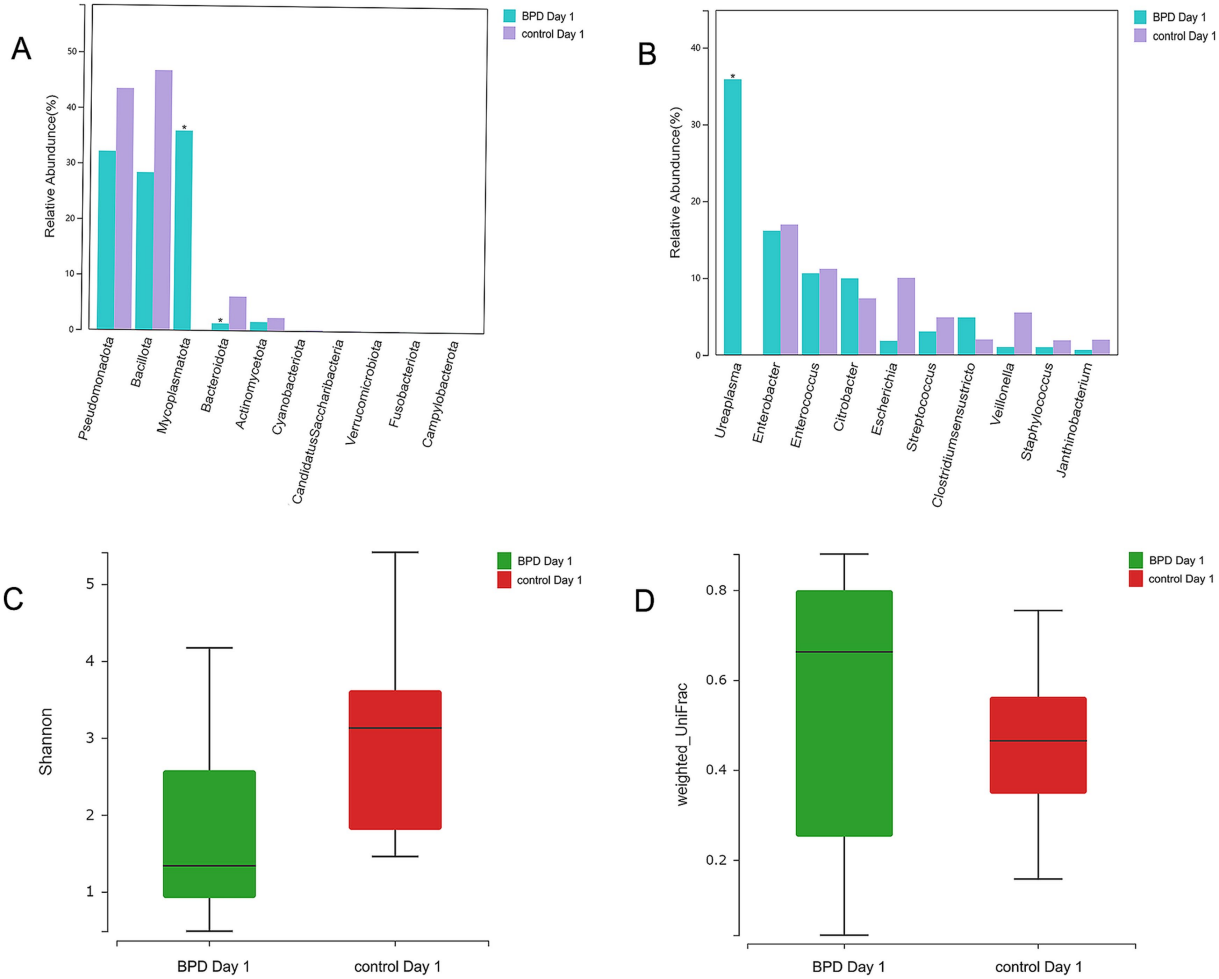
*Mycoplasmatota* and *Ureaplasma* were significantly elevated in the BPD group on day 1 (the BPD group had more cases of maternal chorioamnionitis). Higher abundance of *Mycoplasmatota* and a lower abundance of *Bacteroidota* in the BPD group **(A)**, the BPD group having a significant increase in *Ureaplasma* **(B)**, the BPD group having a significant decrease in Shannon index **(C)**, and the BPD group having a significant increase in beta diversity **(D)**.

**Figure 6:**
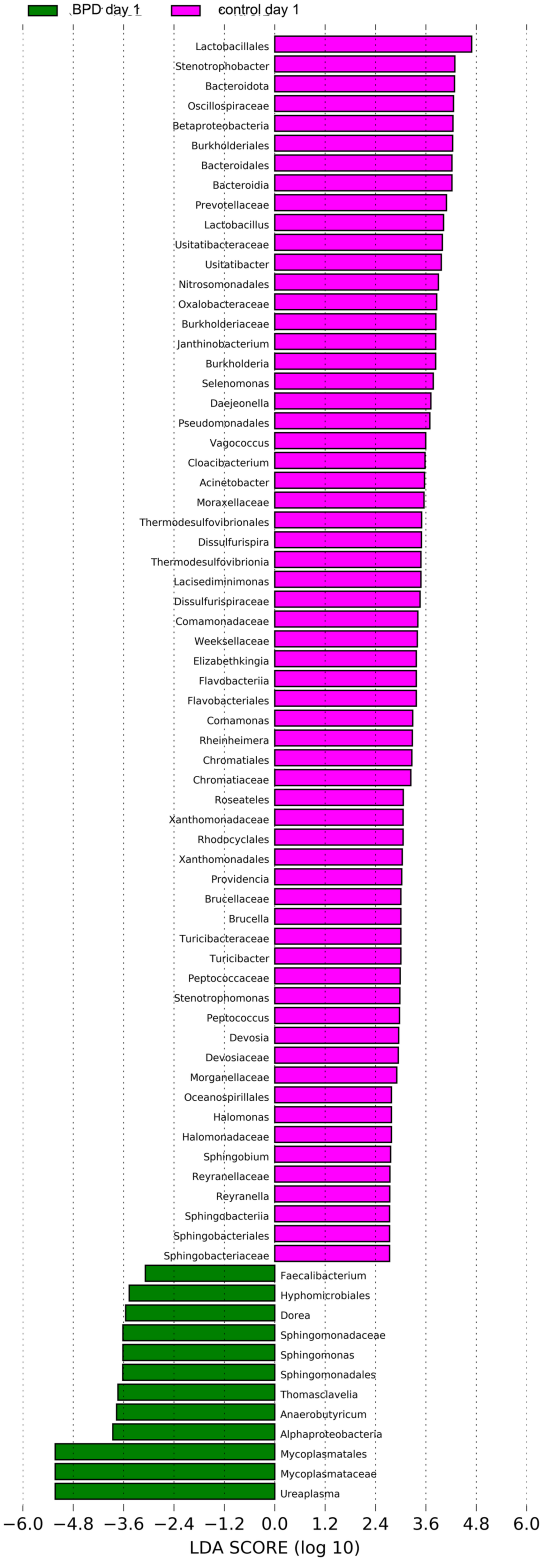
LEfSe showed that *Mycoplasmatales*, *Mycoplasmataceae*, and *Ureaplasma* were the most different species in the BPD group on day 1, and *Lactobacillales*, *Stenotrophobacter*, and *Bacteroidota* were the most different species in the control.

According to comparisons between the two groups, the BPD group showed a significant increase in *Mycoplasmatota* (*p* = 0.027) and a significant decrease in *Bacteroidota* (*p* = 0.011) at the phylum level on day 1 (Figure 5A). A significant decrease was also observed in *Negativicutes*, *Betaproteobacteria*, *Alphaproteobacteria*, and *Flavobacteriia* in the BPD group. The BPD group showed a significant increase in *Ureaplasma* (*p* = 0.037; Figure 5B), where the main species was *Ureaplasma urealyticum* (*p* = 0.037). The BPD group showed a significant decrease in the Shannon index (*p* = 0.035) and a significant increase in beta diversity (*p* = 0.003; Figures 5C,D). The relative abundance of *Bifidobacteria* and *Lactobacillus* was extremely low in both groups. On day 1, the abundance of *Lactobacillus* was significantly lower in the BPD group than in the control group (*p* = 0.03). Linear discriminant analysis (LDA) was performed; the preset LDA value was 2.0, and an absolute LDA score greater than this value indicated a statistically significant difference. Linear discriminant analysis effect size (LEfSe) showed that *Mycoplasmatales*, *Mycoplasmataceae*, and *Ureaplasma* were the most different species in the BPD group on day 1, whereas *Lactobacillales*, *Stenotrophobacter*, *and Bacteroidota* were the most different species in the control (Figure 6).

**Figure 7:**
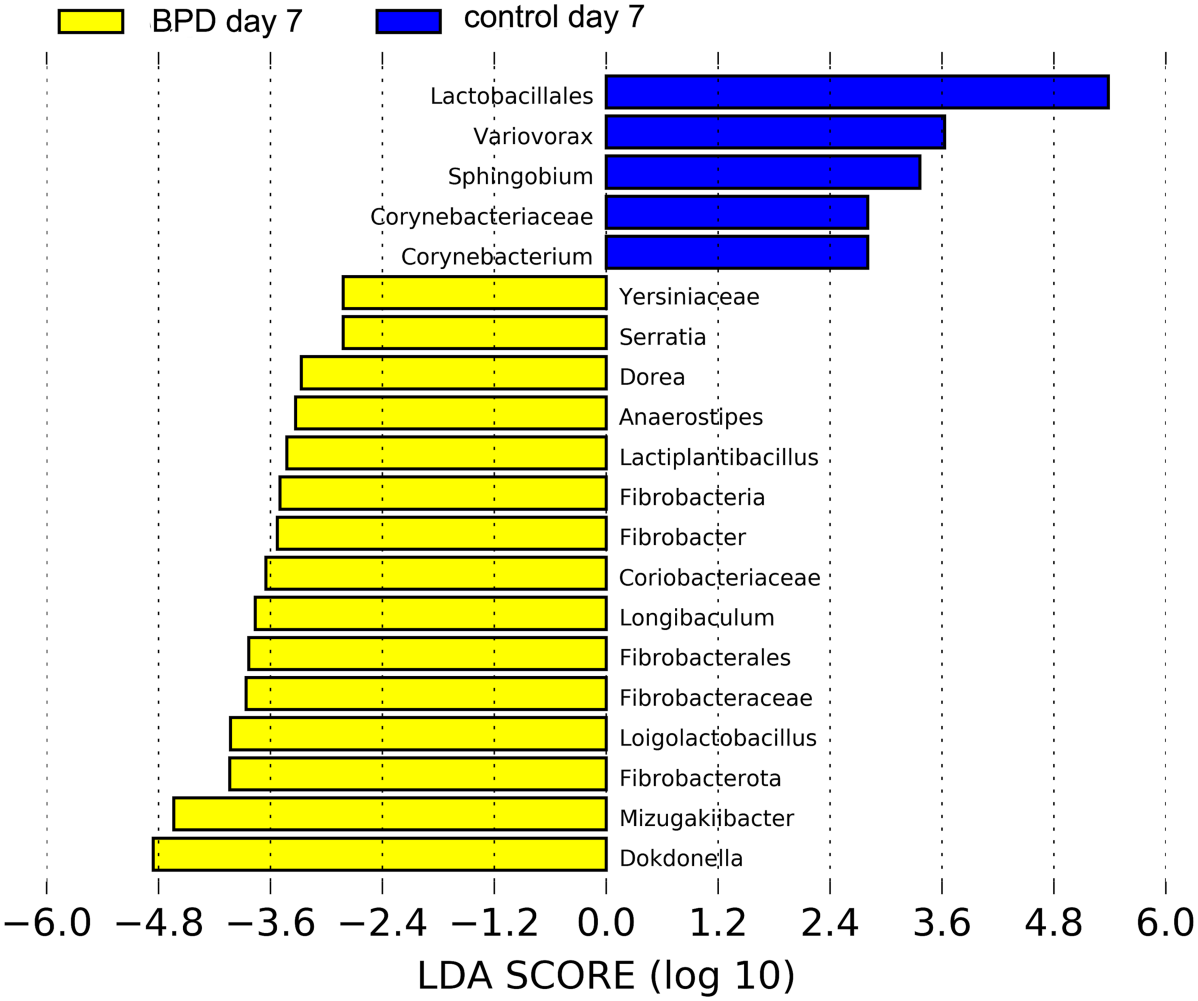
LEfSe showed that *Lactobacillales* was the most different species in the control on day 7 but had a very low relative abundance.

On day 7, no significant difference was observed in all levels of key species between the two groups, nor in the Shannon index (*p* = 0.943) and beta diversity (*p* = 0.968). As indicated by LEfSe, *Lactobacillales was* the most different species in the control but had a very low relative abundance (Figure 7).

**Figure 8:**
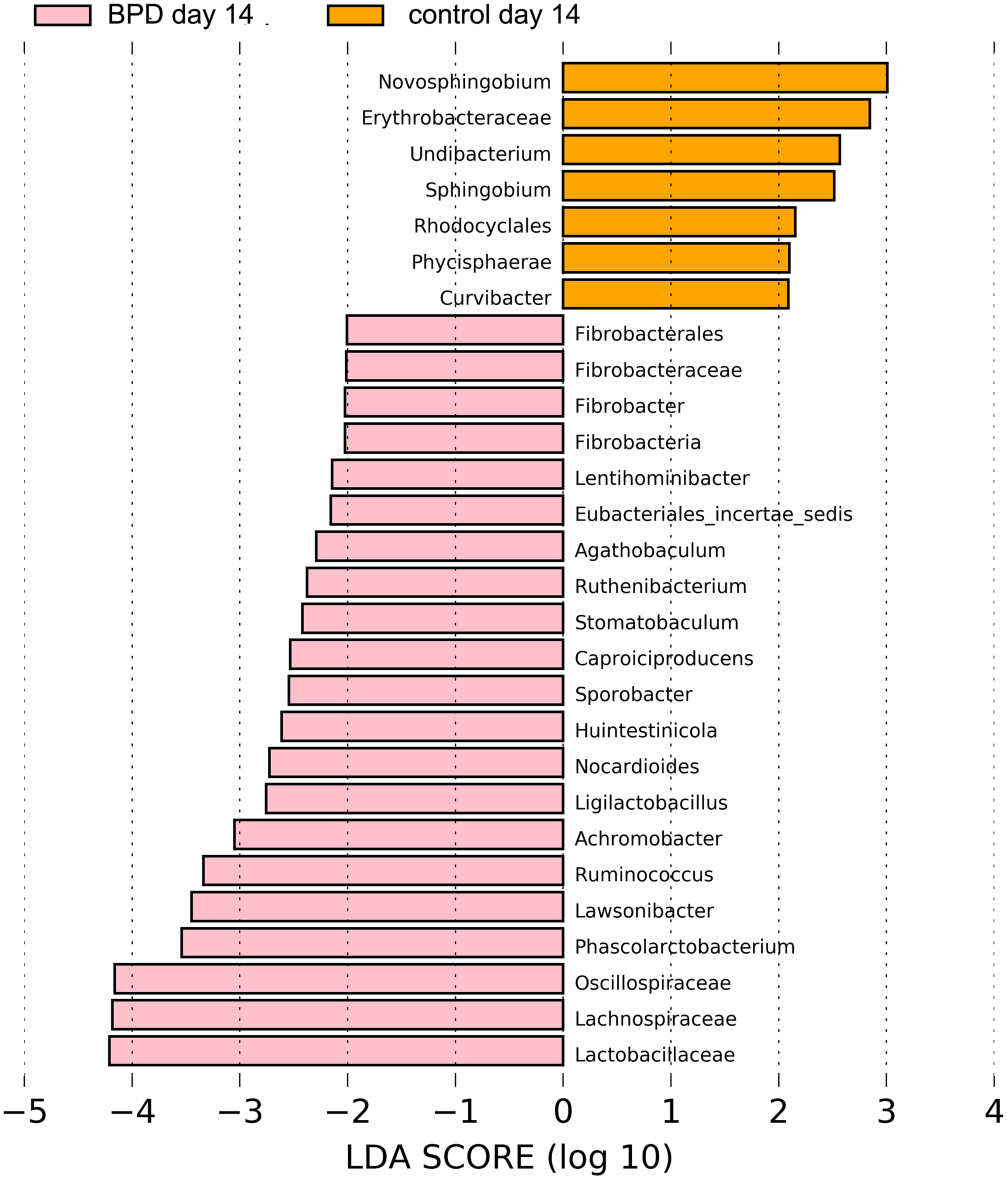
LEfSe showed that *Oscillospiraceae*, *Lachnospiraceae*, and *Lactobacillaceae* were the most different species in the BPD group on day 14, which also had very low relative abundance.

**Figure 9:**
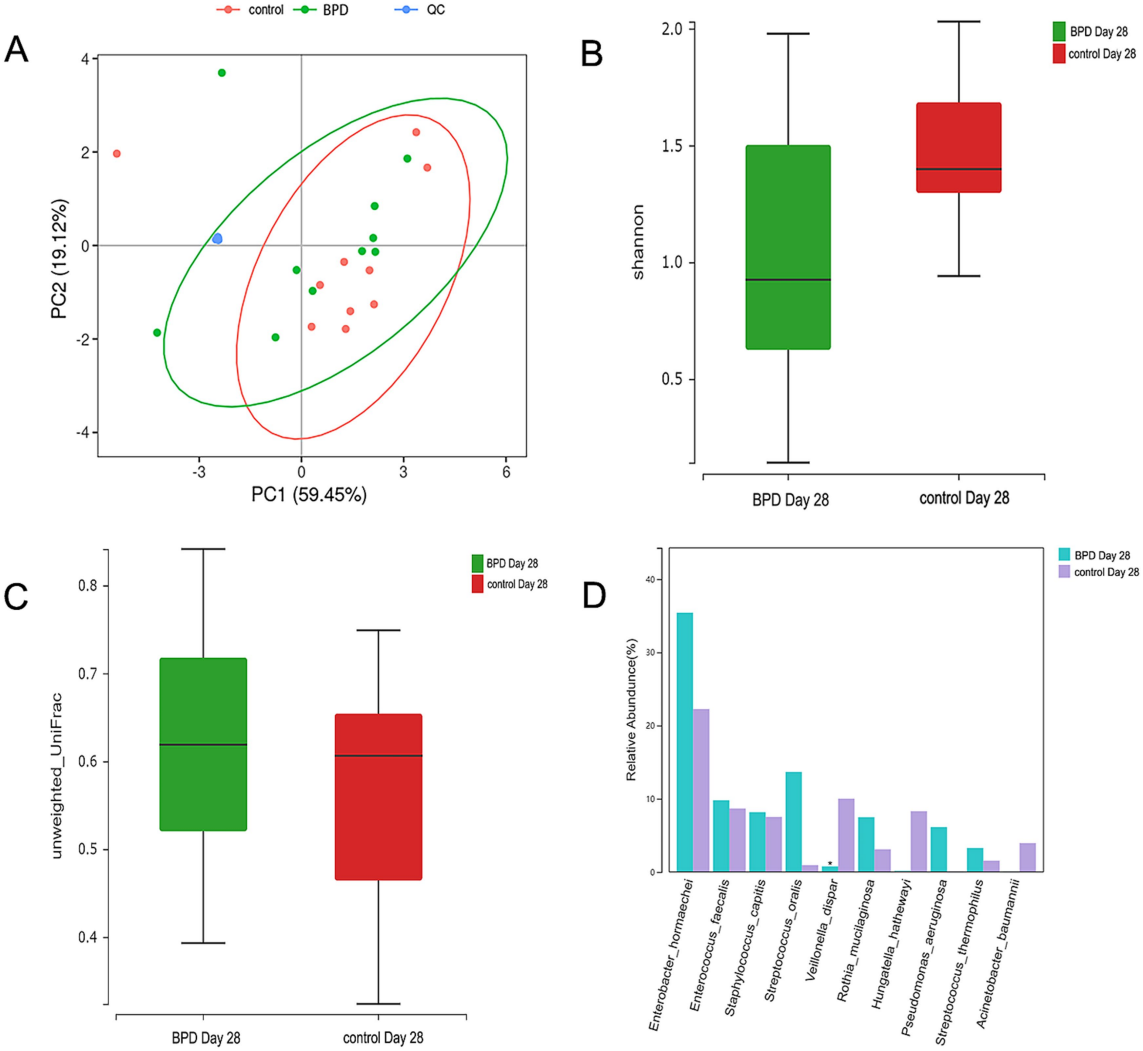
The PCA analysis of SCFAs between the two groups, the ellipse having a 95% confidence interval; each dot indicating a sample, and the different colors of different groups; no difference observed in 7 kinds of SCFAs between the two groups on day 14 **(A)**. Comparisons were made between the two groups on day 28. The Shannon index is lower in the BPD group **(B)**, beta diversity is higher in the BPD group **(C)**, and the BPD group has a significant decrease in *Veillonella dispar* **(D)**.

On day 14, no significant difference was observed in all levels of key species between the two groups, nor in the Shannon index (*p* = 1.000) and beta diversity (*p* = 0.106). LEfSe showed that *Oscillospiraceae*, *Lachnospiraceae*, and *Lactobacillaceae* were the most different species in the BPD group, which also had very low relative abundance (Figure 8). On the same day, ten samples were selected from the BPD group, and ten from the control group and a test was performed on seven kinds of SCFAs within the feces, with no difference in SCFAs content between the two groups, as indicated by the PCA analysis (Figure 9A).

**Figure 10:**
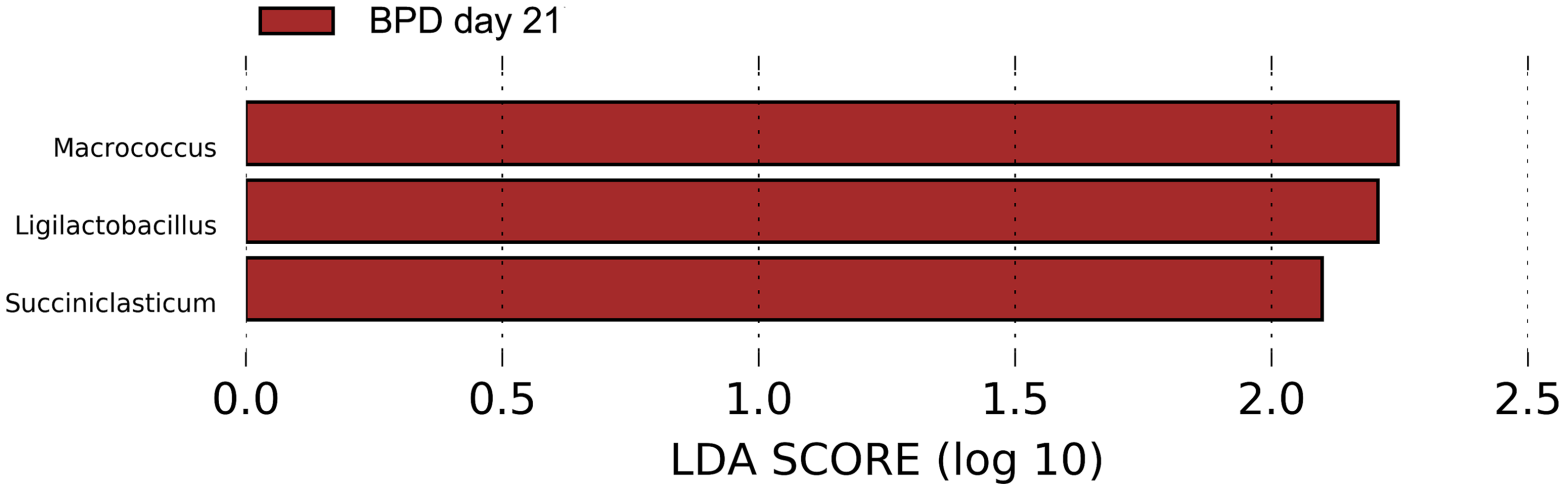
LEfSe showed that *Macrococcus*, *Ligilactobacillus*, and *Succiniclasticum* were the most different species in the BPD group on day 21 but had very low relative abundance.

On day 21, a lower abundance of *Bifidobacterium animalis* (*p* = 0.021) and a higher abundance of *Enterococcus faecalis* (*p* = 0.010) were observed in the BPD group. However, no difference was observed in the Shannon index (*p* = 0.512) or beta diversity (*p* = 0.096) between the BPD and control groups. LEfSe showed that *Macrococcus*, *Ligilactobacillus*, and *Succiniclasticum* were the most different species in the BPD group but had very low relative abundance (Figure 10).

We simultaneously compared and analyzed the differences in the clinical factors between the two groups on days 7, 14, and 21. Except for a lower gestational age (*p* = 0.007), a higher rate of endotracheal intubation (*p* = 0.017), and a lower Apgar score at the time point of 5 min (*p* = 0.008) at birth in the BPD group; however, there was no significant difference in the duration of antibiotics, feeding mode, duration of PN, and duration of non-invasive ventilation on days 7 and 14, but we observed a longer duration of invasive ventilation on days 7 and 14 (*p* = 0.047; *p* = 0.032). We also observed a longer duration of invasive ventilation (*p* = 0.016) and longer administration of antibiotics (*p* = 0.013) on day 21.

**Figure 11:**
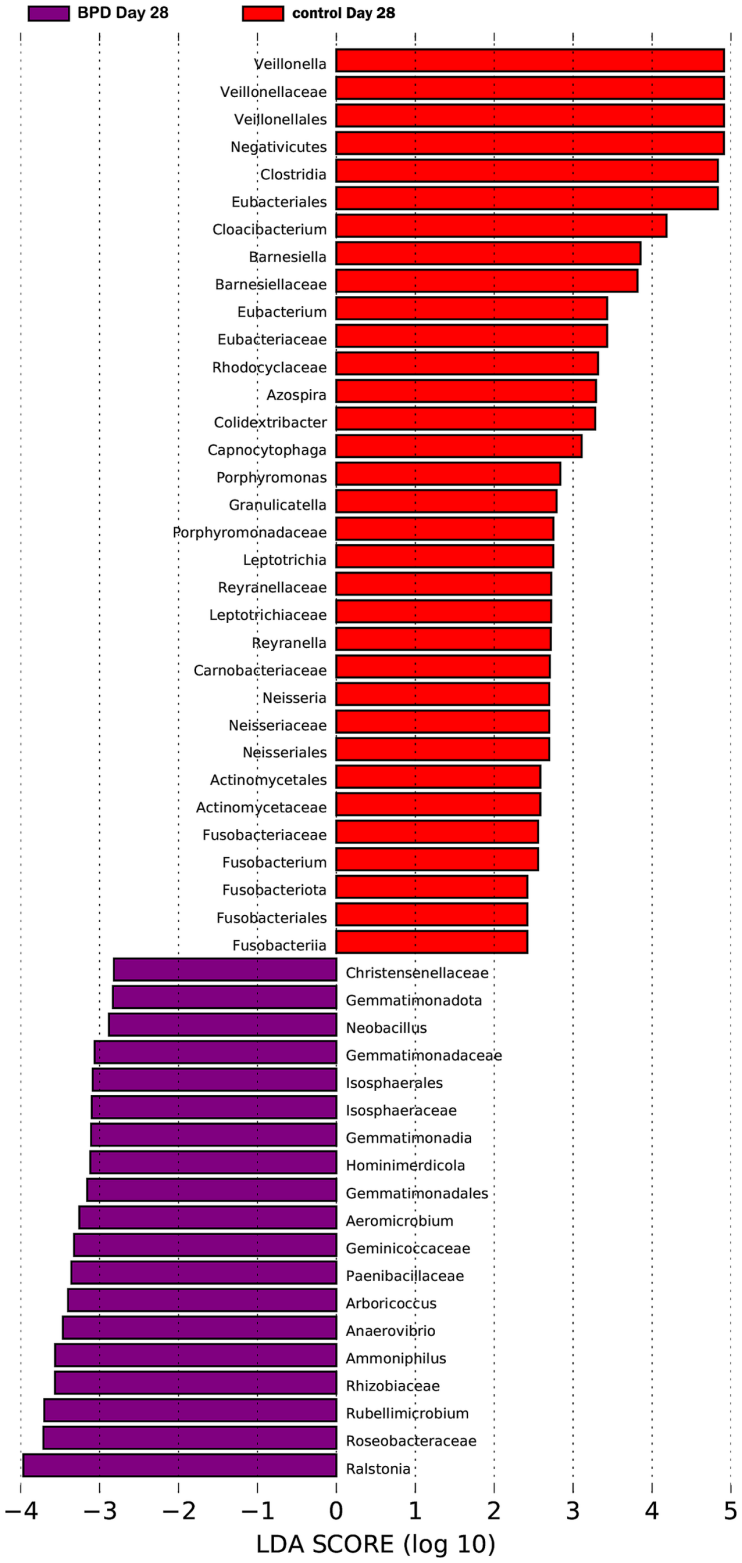
LEfSe LDA diagram showing *Veillonella* as the most different species on day 28.

On day 28, a significant difference was observed in diversity between the two groups. The BPD group exhibited a lower Shannon index, but the difference was not statistically significant (*p* = 0.060), and there was a significant increase in beta diversity (*p* = 0.007; Figures 9B,C). The BPD group had a lower abundance of *Fusobacteriota*, *Clostridia*, *Negativicutes*, and *Eubacteriales* than the control group. We also observed a significant reduction in *Veillonella* in the BPD group (*p* = 0.014), with the main species being *Veillonella dispar* (*p* = 0.023; Figure 9D). We acknowledge that the relatively small sample size (*n* = 15 per group) limited the generalizability of our findings. As indicated in Figure 11 (LEfSe), among the different species of the two groups, *Veillonella* was found to be the most different species. The Kyoto Encyclopedia of Genes and Genomes (KEGG) analysis showed that the function abundance of the microbial community had significant differences between the two groups on day 28, when the BPD group had lower function abundance of “glycan biosynthesis and metabolism” (*p* = 0.028) and higher function abundance of “bacterial infectious diseases” than the control (*p* = 0.018).

### 3.6 Subgroup analysis

In the analysis of the factors affecting microbiota, we did not observe any statistical difference in the respective rates of breast and mixed feeding and cesarean section between the two groups during the period of antibiotic exposure. When we stratified the preterm infants according to the duration of antibiotic use, the period of antibiotic exposure was 1–27 days before day 28 in the BPD group, which was divided into three subgroups: group A of ≤7 days (*n* = 4), group B of 8–14 days (*n* = 5), and group C of >14 days (*n* = 6). The period of antibiotic exposure was 6–14 days in the control group, which was then divided into two subgroups: group A′ of ≤7 days (*n* = 8) and group B′ of 8–14 days (*n* = 7) (Table 3).

**Table 3:** Subgroups stratified according to the duration of antibiotics in the two groups.

Duration	≤7 days	8–14 days	>14 days
BPD	A (*n* = 4)	B (*n* = 5)	C (*n* = 6)
control	A′(*n* = 8)	B′(*n* = 7)	

BPD, bronchopulmonary dysplasia.

Subsequently, we made a comparison of groups A and A′ and observed no significant differences on day 28. We found no significant differences in gestational age (*p* = 0.052), birth weight, feeding mode, delivery mode, or duration of parental nutrition between the two subgroups. However, we observed significant differences in the duration of invasive ventilation (*p* = 0.029) and non-invasive ventilation (*p* = 0.013). When we did the same with groups B and B′ on day 28, intriguingly, we found that the former had a significant decrease in *Veillonella* and *Veillonella dispar* (*p* = 0.009 vs. *p* = 0.006). Moreover, group B had a lower abundance of *Clostridia*, *Negativicutes*, and *Eubacteriales*. In comparison, the clinical data between groups B and B′ were consistent with those between groups A and A′.

## 4 Discussion

During the development of BPD, the intestinal microbiota underwent drastic changes and reduced diversity (Fan et al., 2022). It was previously reported that dysbiosis of the intestinal microbiota increases the level of Lipopolysaccharide (LPS) in preterm infants, which activates inflammatory factors through a signaling pathway. LPS combined with Toll-like receptor (TLR) 4 in alveolar macrophages and alveolar type 2 cells activates the signaling pathway, which leads to an increase in interleukin (IL)-1β, activation of NF-κB, inflammatory cascade, and lung injury (Wedgwood et al., 2020). Intestinal microbiota dysbiosis also damages the mucosal barrier, which causes inflammation, metabolic disorders, and malnutrition, promoting BPD through the gut-lung axis (Yang et al., 2021). The most important factors influencing the intestinal microbiota of preterm infants are gestational age, cesarean section, formula feed, and antibiotics (Cukrowska et al., 2020), which also play a crucial role in BPD (Stricker et al., 2022). We speculated that gut microbiota dysbiosis was not just a concomitant phenomenon; it was closely related to BPD, which might be one of the mechanisms by which perinatal factors enhance BPD. Because not all infants received probiotics within the first 28 days after birth, the intestinal microbiota data were not influenced by probiotics.

A higher abundance of *Mycoplasmatota* was found in the BPD group during the 28 days after birth, which was also significantly higher than that in the control group on day 1. At the species level, a higher abundance of UU was observed in the BPD group, which might be a continuation of intrauterine colonization because the BPD group had more mothers diagnosed with chorioamnionitis and thus more infants diagnosed with UU infection than the control group. These findings suggested that the over-colonization of UU in the gut was intricately linked to both prenatal infection and the development of BPD. UU was a common pathogen of chorioamnionitis, and the proportion of preterm infants exposed to chorioamnionitis had been reported to increase when gestational age decreased, which might reach 80% when gestational age was below 28 weeks (Villamor-Martinez et al., 2019). The evidence that chorioamnionitis increased the risk of BPD might be attributed to the exposure to chorioamnionitis, triggering an inflammatory response in the lungs. This response (Nandakumar and Aly, 2020), releasing many inflammatory mediators, limited the activities of surfactant proteins and vascular endothelial growth factor, resulting in vascular dysplasia and alveolar constriction in the immature lungs (Yang and Dong, 2020).

Our study showed that the relative abundance of *Bacteroidota* was significantly reduced in the BPD group compared to that in the control group on day 1, and it decreased significantly from day 14 to 21 in the BPD group. *Bacteroidota* belonged to anaerobic bacteria, for which the formation of the intestinal anaerobic environment was extremely important (Imoto et al., 2021). Oxygen exposure was usually compounded by the clinical use of supplemental oxygen and mechanical ventilation (Abdelgawad et al., 2023). We observed that the BPD group had a higher rate of endotracheal intubation at birth, and the duration of invasive and non-invasive ventilation before day 28 was longer in the BPD group. The abundance of *Bacteroidota* was also reported to be closely related to gestational age, feeding pattern, and antibiotic use (Tanaka et al., 2009). In our study, the BPD group was characterized by lower gestational age and longer antibiotic treatment. LPS produced by *Bacteroidota* normally had no stimulating effect on immune cells but might have an antagonistic effect on LPS produced by *Proteobacteria* (Jacobson et al., 2018). Some researchers found that the levels of IL-4 and IL-13 increased as the abundance of *Bacteroidota* increased (Imoto et al., 2021; O'Dwyer et al., 2018), and the production of SCFAs reduced with a decrease in *Bacteroidota* (Murugesan et al., 2017). *Bacteroidota might* affect lung development via the mechanisms described above.

In the BPD group, *Veillonella* decreased significantly from days 7 to 28 and was significantly lower than that in the control group on day 28, with the main species being *Veillonella dispar*. *Veillonella*, a gram-negative anaerobic micrococcus, belongs to the commensal microbiota that is distributed in the oropharynx, respiratory tract, and digestive tract. *Veillonella* formed biofilm and interacted with host cells or other microorganisms and produced acetate and propionate via fermentation, having a positive effect on immune balance and other health outcomes (Zhang et al., 2023). *Veillonella* enhanced the immunomodulatory effect in early life, exerting a protective effect on atopic wheezing in childhood (Arrieta et al., 2015), and could convert human milk oligosaccharides, increasing the content of lactate, acetate, and propionate (Button et al., 2023). *Veillonella dispar*, containing nitrate reductase, had strong nitrate reduction activity and reduced nitrate to nitrite, further synthesizing nitric oxide (NO) (Kapil et al., 2020). Researchers found that nitrate reductase activity was significantly lower in preterm infants with BPD than in controls, indicating that *Veillonella* influenced the development of BPD through nitrate reductase activity and the NO signaling pathway (Gentle et al., 2021; Piersigilli and Bhandari, 2020).

Breast milk was reported to protect against BPD by influencing microbiota formation and regulating inflammatory responses (Piersigilli et al., 2020). In the feces of exclusively breastfed newborns, *Veillonella* was enriched, the production of SCFAs increased (Wood et al., 2021), and the most abundant genera in the intestinal microbiota of breastfed preterm infants were *Veillonella* and *Escherichia/Shigella* (Wang et al., 2020). In our study, the BPD group had a lower rate of breastfeeding and mixed feeding than the control group; however, the difference was not statistically significant. We observed a significant decrease in *Veillonella* on day 28 in the BPD group compared to that in the control group, but also a significant decline in both *Veillonella* and *Escherichia* from day 1 to 28 in the BPD group.

A study indicated that 78.6% of very low birth weight preterm infants received antibiotics early in life (Flannery et al., 2018). During the 28 days after birth in our study, all infants received antibiotics, and the BPD group had a longer duration. Newborns exposed to antibiotics had gut microbiota diversity reduced (Zimmermann and Curtis, 2020) and experienced a decrease in the abundance of *Veillonella* (Fu et al., 2020). In terms of infants treated with antibiotics, the colonization of *Bacteroidota* was delayed for 3 months (Tanaka et al., 2009), and antibiotic exposure was associated with a reduction in SCFAs in preterm infants (Jia et al., 2020). Additionally, it was confirmed that there was an increase in *Enterobacter* during the second and third weeks of life among preterm infants who used antibiotics in the first week after birth (Greenwood et al., 2014). In the BPD model of mice, intestinal microbiota dysbiosis induced by broad-spectrum antibiotics participated in the pathogenesis of BPD by increasing the pulmonary inflammatory response (Ran et al., 2021). Antibiotic exposure led to the infant’s intestinal and lung microbiota dysbiosis, aggravating hyperoxia-induced alveolar and angiogenic damage (Chen et al., 2021). Antibiotics were a prominent factor influencing intestinal microbiota and BPD development. Prolonged use of antibiotics in very low birth weight preterm infants increases the risk of BPD (Cantey et al., 2017). We observed not only a lower abundance of *Veillonella* on day 28 and a continuous decreasing trend of *Bacteroidota* but also a higher abundance of *Enterobacter* in the BPD group.

In our study, the rate of cesarean section in the BPD group was close to that in the control group, indicating that cesarean section was not involved in the differences in microbiota.

KEGG analysis revealed that the BPD group had a lower function abundance of “glycan biosynthesis and metabolism” and a higher function abundance of “bacterial infectious diseases” than the control group. As shown in Table 2, more nosocomial infections were observed in the BPD group. These changes appeared to be closely associated with a decrease in *Veillonella*. Zhang et al. have demonstrated the critical role of *Veillonella dispar* in carbon metabolism, glycolysis, and gluconeogenesis (Zhang et al., 2023). As mentioned above, *Veillonella* could form biofilm, interact with host cells or other microorganisms, produce acetate and propionate by fermentation, and convert human milk oligosaccharides, having a positive effect on immune balance (Zhang et al., 2023; Button et al., 2023). To our knowledge, abnormal glycan biosynthesis and metabolism were closely associated with BPD (Fan et al., 2022), and infection and inflammatory responses represented critical components in the pathogenesis of BPD (Thébaud et al., 2019; Martin et al., 2025).

SCFAs, including formate, acetate, propionate, and butyrate, were mainly produced from the catabolism of carbohydrates and cellulose by anaerobic bacteria, including *Bacteroidota* and *Veillonella* (Machado et al., 2021). Acetate was demonstrated to be protective against BPD in the mouse model; the mice treated with acetate had a lower level of IL-1β, IL-18, TNF-α, caspase-1, and NLRP3, respectively, while having a higher level of GPR43 (Zhang et al., 2021). Butyrate and propionate not only promoted the activity of nitric oxide synthase but also inhibited the expression of NF-κB in lung macrophages, thereby reducing inflammation (Park et al., 2019). Researchers have found that SCFAs played a certain role in affecting bone marrow hematopoiesis and promoted the differentiation of bone marrow precursor cells to an anti-inflammatory orientation, thereby reducing lung injury (Dang and Marsland, 2019). In our study, we tested the SCFAs in the two groups on day 14 and found no significant differences between the two groups, which might be due to the absence of key species of microbiota. Regrettably, because of the limitation of only reserving fecal samples on postnatal day 14, we were unable to collect specimens at other time points (including days 1 and 28) for SCFA measurements. We believed that the lower abundance of *Bacteroidota* in the BPD group on day 1 and the continuous decline of *Veillonella* in the BPD group would decrease the production of SCFAs, influencing the development of BPD. Furthermore, the antagonistic effect of *Bacteroidota* on LPS produced by *Proteobacteria* and the anti-inflammatory activity of *Bacteroidota* were also involved in the mechanism of lung development (Jacobson et al., 2018; O'Dwyer et al., 2018).

Although we believed that SCFA levels might differ between the two groups on day 28, because of the missing relevant data, we acknowledged the limitations of the conclusions. In future studies, we plan to simultaneously analyze gut microbiota and SCFA in fecal samples and clarify their relationships.

According to the duration of antibiotics, we stratified the preterm infants and performed subgroup analyses, finding a significant difference in *Veillonella* and *Veillonella dispar* between groups B and B′. From the subgroup clinical data compared, we observed significant differences in the duration of invasive and non-invasive ventilation. In contrast, we did not observe the difference in the microbiota between groups A and A′, although the clinical data were consistent with those between groups B and B′, except for the duration of antibiotics. Undoubtedly, as an anaerobic bacterium, the growth of *Veillonella* was constrained by hyperoxia. Moreover, antibiotic treatment could lead to a significant reduction in the abundance of *Veillonella* (Fu et al., 2020), and the resultant microbiota dysbiosis aggravated the hyperoxia-induced alveolar and angiogenesis damage (Chen et al., 2021), increasing the risk of BPD (Cantey et al., 2017). Based on this evidence, we believe that the differences in microbiota between groups B and B′ were due to the combination of hyperoxia and a longer duration of antibiotics.

As a commensal bacterium, the decrease in *Veillonella* might promote BPD through the disruption of immune balance, reduction of nutrient absorption, decline of SCFAs, increase in bacterial infection, and reduction of NO.

As shown in Table 2, there might be correlations between BPD and other clinical outcomes; however, the intrinsic mechanism and involvement of intestinal microbiota need to be explored in further studies.

There were obvious differences between the two groups on day 1, which indicated that the microbiota was colonized in utero and that the microbiota influenced lung development before birth. The differences in microbiota decreased over time, with no significant differences in key species observed on days 7 and 14, and no difference in SCFAs was observed on day 14. This suggests that the postnatal influence of prenatal factors on the microbiota diminished with time, whereas with the implementation of clinical therapy, postnatal factors exerted a subsequent and continuous effect on the gut microbiota and lung development; hence, the development of new differences on day 28. The difference in *Veillonella* on day 28 aligned with the recent findings reported by Thatrimontrichai et al. (2025).

In summary, there was a higher abundance of *Enterobacter* and *Ureaplasma* and a lower abundance of *Bacillota* in the BPD group than in the control group during the postnatal period of 28 days. The BPD group possessed a higher abundance of UU and a lower abundance of *Bacteroidota* than the control group on day 1. The differences in intestinal microbiota were reduced on days 7 and 14, and no difference in SCFAs existed on day 14. New differences emerged over time, with a significant decrease of *Veillonella dispar* in the BPD group than in the control group on day 28, which showed a continuous decline in the BPD group over time.

As the limitations of our study, the sample size was small, thus limiting the statistical power of our results; oral and pulmonary microbiota were not studied simultaneously, which were potentially associated with BPD; such small amounts of microorganisms as viruses and fungi were not analyzed; we could not entirely avoid the impact of infections on the gut microbiota, however, in our study, none of the preterm infants experienced local gut infections, and the nosocomial infections that did occur happened after day 28; our exploration of targeted and untargeted metabolomics remained quite superficial because the preliminary data did not provide strong indications, mainly due to issues with sample size and stool specimens. Therefore, as a preliminary exploratory study, our next step will be to further investigate the relationship between metabolomics and BPD.

## 5 Conclusion

Intestinal microbiota dysbiosis existed in very preterm infants with BPD. The intestinal microbiota of very preterm infants with BPD had abnormal composition and evolution, which might enhance systemic and pulmonary inflammatory responses, impeding lung development. The increased abundance of UU on day 1 and the decrease of *Veillonella dispar* on day 28 might increase the risk of BPD.

## Data Availability

The original contributions presented in the study are publicly available. This data can be found at: https://www.ncbi.nlm.nih.gov, accession number PRJNA1129886.
